# First Clinical Implementation of Step-and-Shoot Proton Arc Therapy for Head and Neck Cancer Treatment

**DOI:** 10.1016/j.ijpt.2025.100749

**Published:** 2025-04-20

**Authors:** Peilin Liu, Xiaoda Cong, Jian Liang, Xiangkun Xu, Weili Zheng, Craig Stevens, Rohan Deraniyagala, Xiaoqiang Li, Xuanfeng Ding

**Affiliations:** Department of Radiation Oncology, Corewell Health William Beaumont University Hospital, Royal Oak, MI, USA

**Keywords:** Step-and-shoot proton arc therapy, Neck-neck cancer, Adaptive planning

## Abstract

**Purpose:**

Dynamic Spot-scanning Proton Arc (SPArc_-Dynamic_) therapy has gained attention for enhancing dosimetric plan quality. However, its full clinical implementation remains under development. As an interim milestone, we developed step-and-shoot arc therapy (SPArc_-step&shoot_) for head-neck cancer treatment.

**Patients and Methods:**

An in-house spot and energy-layer sparsity optimization algorithm was integrated into a clinical treatment planning system. The algorithm prioritized higher MU-weighted energy layers and spots to ensure delivery efficiency and superior plan quality while meeting machine requirements (≥0.02MU/spot). A Dynamic SPArc simulator calculated delivery times, and a machine-learning-based synthetic CT(synCT) platform monitored dose robustness. In June 2024, a head-neck cancer patient with parotid gland malignancy was treated using SPArc_-step&shoot_ (6600 cGy[relative biological effectiveness] in 33 fx) with 9 static fields at 20-degree intervals. Comparative plans (SFO-IMPT, SPArc_-Dynamic_) were evaluated for dose metrics, delivery times, and adaptive planning.

**Results:**

SPArc_-step&shoot_ and SPArc_-Dynamic_ showed similar target coverage and organ-at-risks sparing, and the plan quality is superior to the 3-field SFO-IMPT in the brainstem, oral cavity, and spinal cord sparing. The simulated continuous arc delivery time is 15.9, 6.32, and 4.31 minutes for SPArc_-step&shoot,_ SFO-IMPT, and SPArc_-Dynamic_, respectively. The actual recorded average treatment delivery time for SPArc_-step&shoot_ in 33 fx is 16.7 ± 1.56 minutes. QA-CT and synCT showed a similar target coverage degradation and perturbation, and a replan was initiated.

**Conclusion:**

The SPArc_-step&shoot_ therapy was successfully implemented in the clinical settings, and first patient was successfully treated between June and August 2024. The synCT platform serves a critical role in the daily monitoring process as SPArc_-Dynamic_ might be more sensitive to the patient geometry changes in HNC treatment.

## Introduction

Dynamic Spot-scanning Proton Arc (SPArc_-Dynamic_) therapy has emerged as a high interest in the field of particle beam therapy.^1^ With its capacity to incorporate an additional degree of freedom through arc trajectory via optimization and treatment delivery, it not only enhances the dosimetric plan quality for a broad range of clinical indications—including head and neck (HN), brain, lung, liver, breast, and prostate cancers—but also effectively reduces delivery time and improves treatment efficiency.^2^, ^3^, ^4^, ^5^, ^6^ This technological advancement opens a new era of particle beam therapy, allowing for superior conformality in target dose distribution, more flexibility in linear-energy transfer modulation, which potentially improves tumor control while minimizing the dose delivered to surrounding healthy tissues compared to the state-of-the-art technique, multifield intensity modulated proton therapy (IMPT).^7^ Since its initial concept was published in 2016, SPArc has transitioned from theoretical frameworks to experimental validation. Key milestones include the first experimental demonstration of SPArc delivery by Li et al,^8^ which established the feasibility of integrating spot-scanning with dynamic gantry rotation. This work enabled continuous energy-layer modulation during gantry motion.^8^ A subsequent breakthrough occurred in 2024 with the first preclinical delivery of DynamicARC therapy, which marked a critical advancement by achieving fully continuous proton arc delivery—synchronizing gantry rotation, energy switching, and spot scanning in real time.^9^ Despite its immense potential and clinical benefits, the clinical implementation of SPArc_-Dynamic_ is still undergoing development, with a significant focus on regulatory clearance, system engineering challenges, treatment planning optimization algorithm, oncology information system (OIS) integration, and quality assurance (QA) devices to ensure precise, safe, and robust clinical implementation.^8^

As an interim step toward achieving full SPArc_-Dynamic_ clinical implementation, the development of step-and-shoot Spot-scanning proton arc therapy (SPArc_-step&shoot_) presents a practical milestone. This approach simplifies the delivery mechanism, which is compatible with most clinical software and platforms while retaining many of the dosimetric benefits associated with SPArc_-Dynamic_, except for the treatment delivery efficiency. SPArc_-step&shoot_ represents a feasible solution to bridge the gap between conventional static proton therapy and the more complex dynamic arc systems, such as the IBA Proteus ONE DynamicARC module, making it a valuable temporary method for treating clinically challenging cases such as head-neck cancer.

Recent research efforts have demonstrated the potential of proton arc therapy to enhance dosimetric gains and reduce toxicity in HN cancer treatments. SPArc has been associated with reduced toxicity in oropharyngeal cancer patients, underscoring its potential clinical benefits.^10^ For bilateral HN cases, studies exploring partial and full arcs with DynamicARC techniques have highlighted improved target coverage and organ-at-risk (OAR) sparing, though technical challenges remain in clinical implementation.^11^ The feasibility of SPArc therapy in bilateral HN cancer further supports its dosimetric advantage over IMPT, particularly in minimizing doses to critical structures.^12^ Additionally, de Jong et al^10^ show that proton arc therapy provides potential clinical outcomes for oropharyngeal cancer via a model-based approach, compared to IMPT and VMAT.

In this study, we focus on the clinical application of SPArc_-step&shoot_ therapy for the head-neck cancer patient population. By comparing its dosimetric performance, delivery time, and interfractionation robustness with conventional IMPT and SPArc_-Dynamic_ modalities, we aim to evaluate its potential as an interim solution and to pave the way for advancing toward SPArc_-Dynamic_ implementation. This work also contributes to the growing body of evidence supporting SPArc therapy and its role in providing highly conformal and effective treatment options for a diverse range of cancer patients.

## Methods and materials

### Case description of clinical detail

In April 2024, a 46-year-old female was diagnosed with left parotid adenoid cystic carcinoma. She underwent surgical resection and was found to have a positive margin on her facial nerve. Given the neurotropic nature of this tumor, radiation is needed and has to target the nerve pathways entering the skull base. For a young woman who can be cured and has long-life expectations, proton beam therapy is preferred to deliver high-dose radiation to the base of the skull while reducing the risk of secondary malignancy. Additionally, as the target is in proximity to the brainstem and spinal cord, SPArc_-step&shoot_ is suggested to provide a better dose conformity over IMPT. We elected to use SPArc_-step&shoot_ therapy instead of IMPT in this clinical case. Target delineation included a 6600 cGy(relative biological effectiveness [RBE]) Clinical Target Volume (CTV), including the parotid bed and facial nerve to the skull base. Additional CTVs for 5940 cGy(RBE) and 5280 cGy(RBE) were used to cover more proximal portions of the innervating nerves to the skull base and their entrance to the brain.

### Treatment planning and dosimetric plan quality comparison

The SPArc_-step&shoot_ plan was generated through an in-house developed optimization algorithm, which is implemented in the Raystation (RayResearch Laboratories, Stockholm, Sweden) through scripting.^1^ The SPArc_-step&shoot_ algorithm iteratively selects the higher MU-weighted energy layer and spots to ensure (1) the efficiency of the treatment delivery of SPArc_-step&shoot_ therapy and (2) superior plan quality while meeting the minimum-MU per spot machine requirement (0.02MU per spot, IBA Proteus ONE). The SPArc_-step&shoot_ plan was generated with 9 static fields with evenly distributed gantry angles (20 degrees apart).^13^, ^14^

**Figure 1:**
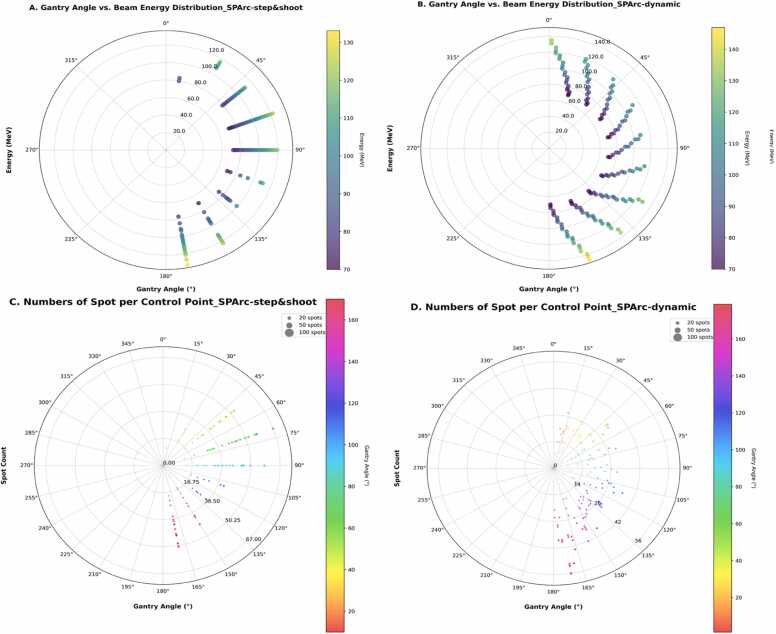
Angular distribution of gantry angle versus selected beam energy and number of protons per control point for SPArc_-step&shoot_ and SPArc_-dynamic._

As a comparison, Three-field Single Field Optimization Intensity Modulated Proton Therapy (SFO-IMPT) and SPArc_-Dynamic_ plans are generated as a comparison.^1^ The SPArc_-Dynamic_ plan with 2.5 deg sampling frequency and half arc (0-180°) uses the SPArc_-seq_ optimization algorithm.^13^ The details of plan parameters could be found in Table S1 and Figure 1.

RBE 1.1 was applied for proton dose calculations. All the modalities of plans are applied to robustness optimization with 3.5% range uncertainties and 3 mm setup uncertainties.^15^ Dose calculations were performed using the Monte Carlo method with a dose grid resolution of 3 mm × 3 mm × 3 mm.

The prescription dose is 66 Gy in 33 fractions. Dose metrics and dose-volume histogram analysis, including CTV target coverage and OAR sparing, which can be found in Table 1, are used to compare the plan quality among the 3 planning groups.

**Table 1:** Dosimetric parameter comparison between SFO-IMPT, SPArc_-step&shoot_, and SPArc_-dynamic_ plan.

Organs at risk (OAR)	SFO-IMPT	SPArc_-step&shoot_	SPArc_-dynamic_
CTV6600(%)	99.80	99.73	99.60
CTV5940(%)	98.73	99.76	99.65
CTV5280(%)	99.76	100.00	99.87
Brainstem Dmax(cGy)(RBE)	3666	3315	3104
Optical chiasm Dmax(cGy) (RBE)	430	207	189
Oral Cavity Dmean(cGy) (RBE)	18	5	5
Spinal canal Dmax(cGy) (RBE)	1783	188	328

Abbreviations: CTV, clinical target volume; IMPT, intensity modulated proton therapy; RBE, relative biological effectiveness.

### Treatment interfractionation robustness evaluation

During the treatment course, an in-house developed machine-learning-based synthetic CT (synCT) was implemented in the clinic, which generates synCT based on the daily cone beam computed tomography (CBCT) for offline daily dose reconstruction and monitoring purposes for SPArc_-step&shoot_.^16^ With the observation of the patient geometry changes and dose perturbations on the daily synCT, a new computed tomography (CT) sim was scheduled, and an adaptive plan was generated in the 13th fx to improve the interfractionation robustness. Daily dose reconstruction was continuously evaluated by assessing target coverage and OAR sparing based on the synCT platform throughout the treatment course.

### Patient-specific SPArc_step&shoot_ plan QA

Patient-specific QA was performed to ensure the accuracy and safety of the SPArc_step&shoot_ plan prior to clinical implementation. QA procedures included measurement-based verification using a phantom setup to validate the dose distribution via 2D ionization chamber array (MatriXXONE, IBA dosimetry). A 3% dose difference and 3 mm distance to agreement were used in the field of gamma index evaluation.

### Treatment delivery time estimation and recording

Prior to the actual treatment, the estimated treatment delivery time among the 3 plans was simulated via a published clinical PTS model, IBA Proteus ONE, and its dynamic arc therapy system controller.^17^, ^18^ The dynamic arc therapy system controller considered not only the static irradiation time but also the gantry mechanical limitations and constraints, for example, 6deg/s max speed and 0.6 deg/s^2^ maximum acceleration or deceleration speed. Total dynamic treatment delivery time includes both continuous arc and step&shoot delivery, which takes into account of the mechanical constraints of the gantry during the simulation, for example, gantry acceleration and deceleration. For the total dynamic treatment delivery mechanism, including SFO-IMPT and SPArc_-step&shoot_ plan, an additional 1.5 minutes per field transfer between the OIS to PTS and treatment field preparation time was estimated. The actual SPArc_-step&shoot_ delivery time throughout the entire treatment course was extracted from OIS for validation purposes.

## Results

### Dosimetric parameter comparison

**Figure 2:**
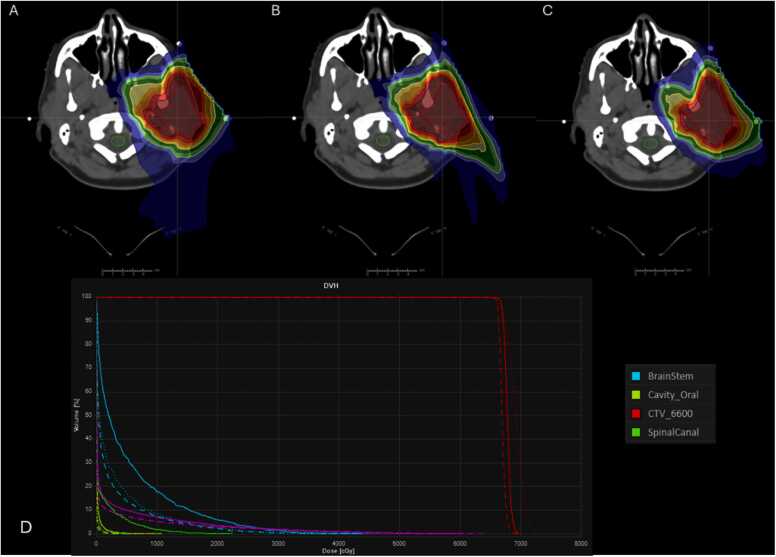
A representative slice of dose distribution comparison from the head-neck cancer patient. (A) SFO-IMPT plan; (B) SPArc_-Dynamic_ plan; (C) SPArc_-step&shoot_; (D) dose-volume histograms (solid line: SFO-IMPT plan; dotted line: SPArc_-Dynamic_ plan; and short dashed line: SPArc_-step&shoot_ plan). Abbreviation: IMPT, intensity modulated proton therapy.

Three modalities showed similar target coverage above 98% of the prescription dose and satisfied the clinical requirement. However, SPArc_-step&shoot_ and SPArc_-Dynamic_ have better protection of OARs, which includes brainstem, brainstem chiasm, and spinal cord sparing. Specifically, the maximum dose to the brainstem was 3666 cGy(RBE), 3315 cGy(RBE), and 3104 cGy(RBE) for SFO-IMPT, SPArc_-step&shoot_, and SPArc_-Dynamic_, respectively. SPArc_-step&shoot_ and SPArc_-Dynamic_ significantly reduced the spinal canal dose from 1783 cGy(RBE) to 188 cGy(RBE) and 328 cGy(RBE), respectively. Additionally, the maximum dose to the optic chiasm decreased from 430 ] cGy(RBE) with SFO-IMPT to 207 cGy(RBE) with SPArc_-Dynamic_ and 189 cGy(RBE) with SPArc-_step&shoot_. Furthermore, there was a modest improvement in the mean dose to the oral cavity, decreasing from 18 cGy(RBE) for SFO-IMPT to 5 cGy(RBE) and 5 cGy(RBE) for SPArc_-step&shoot_ and SPAr_c-Dynamic_, respectively (Table 1 and Figure 2).

### Interfractionation robustness evaluation

**Figure 3:**
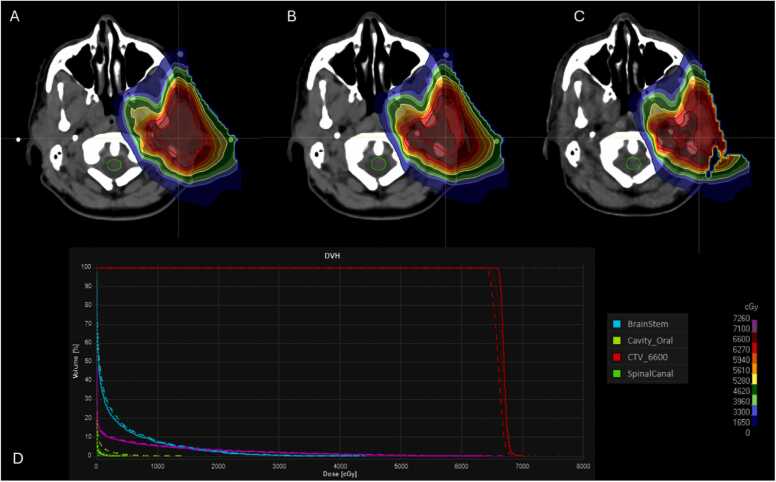
A representative slice of dose distribution comparison from the head-neck cancer patient and among nominal SPArc_-step&shoot_ plan calculation on the initial CT, QA-CT on 11th fx and synCT from CBCT on 11th fx (A) nominal plan on initial CT; (B) nominal plan on QA-CT; (C) nominal plan on synCT; (D) dose-volume histograms (solid line: initial CT; dotted line: QA-CT; and short dashed line: synCT). Abbreviation: CBCT, cone beam computed tomography; QA, quality assurance.

**Figure 4:**
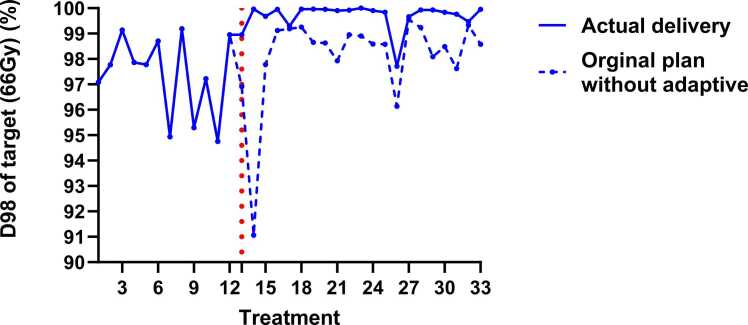
D98 of 33-fraction delivery, the offline adaptive plan has been applied to ensure the target dose coverage from the 13th fraction.

33 sets of synCT have been generated. During the first 12 treatment fractions, all synCTs demonstrated a similar target coverage perturbation and degradation (ranging from 3% to 5% in D98) due to the weight loss and setup uncertainties since the initial CT simulation earlier. As a result, an adaptive plan was initiated at fraction 13 (Figure 3). The adaptive plan improved the target's robust coverage throughout the rest of the treatment course (Figure 4).

### Patient-specific QA for SPArc_step&shoot_ plan and delivery time comparison

The patient-specific QA gamma passing rates at 3%/3 mm criteria exceeded 95% for the SPArc_-step&shoot_ plan, which meets clinical standards for delivery. The simulated total treatment delivery time, which includes gantry rotation and static delivery time, is 15.9, 6.32, and 4.31 minutes for SPArc_-step&shoot_, SFO-IMPT, and SPArc_-Dynamic_ plans based on our IBA Proteus ONE model, respectively (Table 2). The actual recorded time for SPArc_-step&shoot_ across the entire 33 fx is 16.7 ± 1.56 minutes from the OIS.

**Table 2:** Delivery time comparison (simulations and actual recorded treatment time).

Treatment technique	Simulated static irradiation time (min)	Simulated total treatment delivery time (min)	Actual recorded treatment time (min)
SPArc_-step&shoot_	4.01	15.90	16.7 ± 1.56
SFO-IMPT	3.06	6.32	N/A
SPArc_-Dynamic_	4.28	4.31	N/A

## Discussion

We report the first clinical treatment of the SPArc_-step&shoot_ therapy using IBA Proteus ONE integrated with a treatment delivery time simulator^19^ and machine-learning-based syn CBCT offline dose validation platform.^20^ An in-house developed efficient SPArc_-step&shoot_ optimization algorithm was successfully implemented in the clinical TPS through scripting, which effectively reduced the total number of energy layers by a factor of 2.67, the total number of spots by a factor of 2.29, and the treatment irradiation time by a factor of 2.13. (Supplemental Document S1) In addition to the irradiation time, gantry acceleration and deceleration, OIS and patient positioning system (PTS) communication time prolongs SPArc_-step&shoot_ treatment delivery time compared to the SPArc_-Dynamic_ therapy, which is able to deliver the treatment via the continuous gantry or patient position system rotation.

Although dynamic treatment is the ultimate goal for an efficient and broader routine clinical implementation, there are several areas remaining to be developed to ensure a precise and safe clinical implementation of dynamic arc therapy. For example, (1) A new dynamic arc systematic controller is designed to synchronize the irradiation with the gantry rotation. This is a core part of the dynamic arc system to ensure the precise spot irradiation in each gantry angle. (2) A new QA device and procedure to validate dynamic beam delivery accuracy while the gantry continuously rotates. The new QA device and procedure need to validate the actual delivered spot position, MU per gantry angle, and 3D dose distribution from the proton arc therapy; (3) New commission procedures and guidelines are needed for the dynamic proton arc therapy module, including new acceptance criteria and additional validation items. The successful clinical implementation of SPArc_-step&shoot_ therapy in July 2024 not only served as an important interim milestone toward the SPArc_-Dynamic_ therapy but also demonstrated its feasibility for clinical implementation, which is especially important for particle therapy institutions without a dynamic gantry or PBS modules.

On the other hand, SPArc_-step&shoot_ therapy is still a multifield IMPT static treatment delivery technique. Unlike the potential dosimetric perturbations associated with the dynamic treatment delivery mechanism, the static treatment technique has no such concerns.^9^, ^18^ All the existing QA measurement devices, criteria, and OIS integrations are still in place. This compatibility significantly lowers the barrier for clinical adoption, making arc-based treatment more accessible to proton therapy facilities with conventional infrastructure.^21^ However, these benefits come with inherent trade-offs - the technique requires more energy layers and spots compared to SPArc-Dynamic, resulting in longer delivery times that may increase susceptibility to intrafractional motion effects and associated dose delivery uncertainties.^9^, ^10^

Another major finding is that SPArc therapy could be very sensitive to the patient's geometry changes, especially for HNC, due to its characteristics of optimization strategies, where the highly modulated fluence from each control point is generated from the multifield robust optimization.^1^ As some of the control points were coming from the tangent angles, weight loss could lead to a larger target degradation in the presence of daily setup uncertainties. Thus, special care is needed to monitor the patient's weight or geometry changes throughout the treatment course to ensure the initial nominal plan provides a robust target coverage. The in-house developed sync CT platform for offline dose validation served a critical role in assisting the plan adaptation decision. By utilizing synthetic CT, dose perturbation can be quantitatively evaluated in addition to the qualitative check of the patient’s anatomical changes and setup uncertainties, which has demonstrated its clinical importance in the era of proton arc therapy. While synCT derived from daily CBCTs enabled retrospective dose reconstruction and offline monitoring, adaptive planning itself was performed exclusively on CT scans. This hybrid workflow leverages synCT’s capability to detect dosimetric deviations between CT simulations from the initial plan while reserving high-fidelity CT for adaptive replanning. The integration of synCT-based CBCT also offers clinical advantages, including reduced reliance on repeat CT imaging, thereby lowering cumulative patient radiation exposure and extra clinical burden on the CT sim technologists.^22^ However, uncertainties persist regarding synCT accuracy in regions of abrupt tissue and the propagation of CBCT artifacts into synthetic images, which may affect proton range prediction.^23^ Additionally, residual discrepancies between synCT and clinical CT Hounsfield unit calibration necessitate cautious interpretation of absolute dose values.^24^ The knowledge and experience of the patient weight, geometry, and dose monitoring gained from the SPArc_-step&shoot_ therapy can be translated into future clinical implementation of the SPArc_-Dynamic_ therapy.

## Conclusion

The first SPArc_-step&shoot_ therapy was successfully implemented in the clinical PTS to improve the potential clinical treatment outcomes for HNC with a reasonable treatment time. SPArc_-Dynamic_ therapy has the potential to significantly improve the treatment efficiency and ensure a border adaptation of the new technique. On the other hand, the SPArc technique could be more sensitive to the patient weight and geometry changes, and daily offline dose validation is highly recommended to ensure a safe and precise clinical treatment.

## Author Contributions

Conceptualization: **X.D., R.D.**; Data curation: **P.L., X.D.**; Formal analysis: **P.L.**; Investigation: **P.L., X.C., J.L., X.X., W.Z., C.S., R.D., X.L., X.D.**; Methodology: **X.D., P.L.**; Supervision: **X.D., R.D., C.S.**; Writing: **P.L., X.D.**

## Declaration of Conflicts of Interest

The authors declare the following financial interests/personal relationships which may be considered as potential competing interests: Xuanfeng Ding reports financial support was provided by IBA. Xuanfeng Ding reports a relationship with Ion Beam Applications SA that includes funding grants, speaking and lecture fees, and travel reimbursement. Xuanfeng Ding has patent Particle Arc Therapy licensed to IBA. If there are other authors, they declare that they have no known competing financial interests or personal relationships that could have appeared to influence the work reported in this paper.

## Footnotes

Appendix A Supplementary data associated with this article can be found in the online version at doi:10.1016/j.ijpt.2025.100749.
