# Supplementary material for: First Clinical Implementation of Step-and-Shoot Proton Arc Therapy for Head and Neck Cancer Treatment
Source: Int J Part Ther. 2025 Apr 20;16:100749. doi: 10.1016/j.ijpt.2025.100749 (PMC12138570; doi:10.1016/j.ijpt.2025.100749)
Supplement: Supplementary file 1 — Supplementary material [file mmc1.docx]

Supplemental Document

Table S1 Plan parameter comparison between SFO-IMPT, SPArc_-step&shoot_ and SPArc_-dynamic_ plan

| Plan parameter | SFO-IMPT | SPArc_-step&shoot_ | SPArc-_dynamic_ |
| --- | --- | --- | --- |
| Total number of spots | 3026 | 3568 | 3355 |
| Total number of energy layer | 91 | 106 | 152 |
| Gantry angle/range (°) | 50, 90, 180 | 10, 30, 50, 70, 90, 110,130, 150, 170 | 1.3-178.8 |


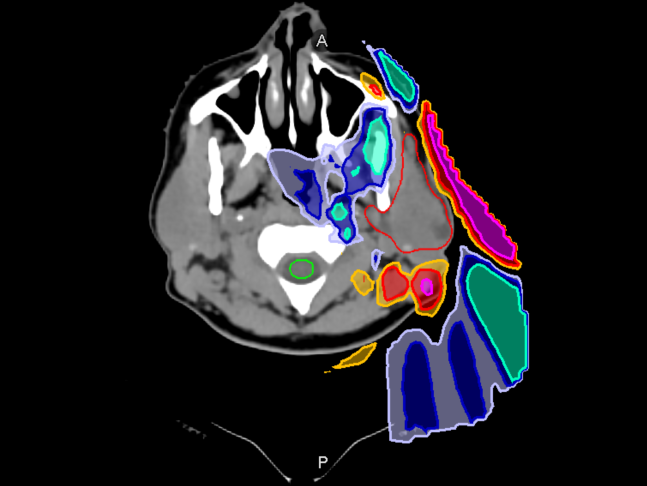

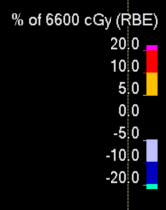


Figure S1. Dose difference between SPArc_-step&shoot_ and SPArc_-dynamic_ plan.


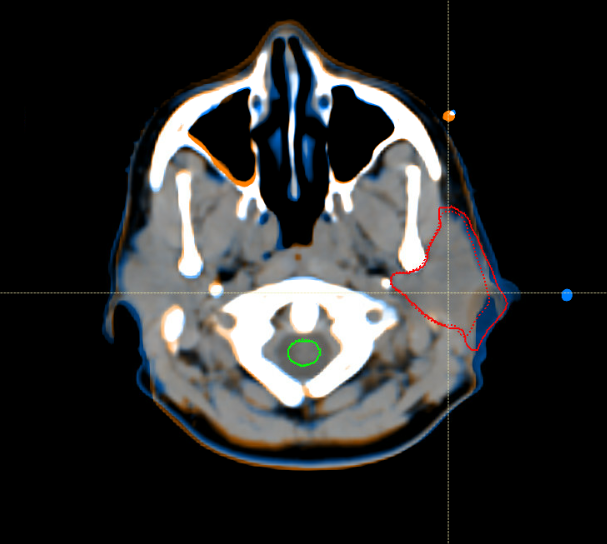


Figure S2. The patient geometry changes between planning CT and re-planning CT. Red solid line: Target in planning CT; Red dash line: Target in re-planning CT.
